# Performance enhancement, elite athletes and anti doping governance: comparing human guinea pigs in pharmaceutical research and professional sports

**DOI:** 10.1186/1747-5341-9-4

**Published:** 2014-02-05

**Authors:** Silvia Camporesi, Michael J McNamee

**Affiliations:** 1 Department of Social Science, Health & Medicine, D6, 3rd floor, East Wing, King’s College London, Strand, London, WC2R 2LS, UK; 2 College of Engineering, Swansea University, Singleton Park, Swansea, SA2 8PP, UK

**Keywords:** Guinea pig, WADA, Research ethics, Sports medicine, Clinical trial, Pharmaceutical research, Visibility, Multiplicity, Consistency

## Abstract

In light of the World Anti Doping Agency’s 2013 Code Revision process, we critically explore the applicability of two of three criteria used to determine whether a method or substance should be considered for their Prohibited List, namely its (potential) performance enhancing effects and its (potential) risk to the health of the athlete. To do so, we compare two communities of human guinea pigs: (i) individuals who make a living out of serial participation in Phase 1 pharmacology trials; and (ii) elite athletes who engage in what is effectively 'unregulated clinical research’ by using untested prohibited or non-prohibited performance enhancing substances and methods, alone or in combination. Our comparison sheds light on norms of research ethics that these practices exacerbate with respect to the concepts of multiplicity, visibility, and consistency. We argue for the need to establish a proper governance framework to increase the accountability of these unregulated research practices in order to protect the human guinea pigs in elite sports contexts, and to establish reasonable grounds for the performance enhancing effects, and the risks to the health of the athlete, of the methods and substances that might justify their inclusion on the Prohibited List.

## Introduction

Nearly 25 years ago, von Ammon and Wettstein asserted that “Bioethics has paid little attention to the issues raised by health and medical care in athletic competition” [1]. While there has been recent discussions of this nexus, the situation has not developed far [2,3]. In this paper we wish to help to redress this tendency of the bioethical discourse, and address a critical but neglected issue at the intersection of medical ethics and sport ethics: how performance enhancing technologies are introduced in competitive athletics, and what implications they have on the athletes-subjects. We argue that the introduction of performance enhancing technologies in the practice of professional sports amounts to unregulated clinical research, and that athletes are vulnerable research subjects, or 'guinea pigs’. We position our discussion in relation to other problematic practices of participation in research, such as healthy individuals who make a living out of serial participation in Phase 1 trials in the pharmaceutical research context. Our analysis points to the exacerbations of classical problems of research ethics when translated to the professional sports context and the pharmaceutical research context, and to the necessity of establishing a proper framework for research on performance enhancing agents.

## Athletes as guinea pigs?

Elite sport is an arena where sports medicine and sports science teams have been keen to exploit biomedical and biotechnological innovations regarding injury, treatment of injury and health optimization, irrespective of the epistemic support for their practices. The motivational force of elite sports status has led to the proliferation of innovative interventions that have in turn led to three leading sports medicine experts to wonder whether the field has more than its fair share of “snake oil” salesmen [4]. Examples range from the more to less credible, and include the use of platelet rich plasma to enhance recovery (despite its disputed evidence base) [5], or pre-emptive surgery (as in Tommy John elbow tendon replacement mistakenly thought to enhance baseball pitching performance by eager excessively enthusiastic parents) [6]. More recent discussion includes examples of athletes who might experiment with the latest generation of gene transfer techniques to enhance their performances [7].

Given the power-saturated contexts of elite sport, athletes often find themselves vulnerable actors in the nexus of networks between sport and medicine [8]. To the best of our knowledge, King and Robeson (2007) were the first to highlight the problematic issue of how the introduction of broadly conceived performance enhancing technologies in competitive athletics constitutes unregulated clinical research [8]. King and Robeson comment on their position at the cutting edge of science and technology defining athletes as “unwitting or unwilling research subjects”, or “guinea pigs” ([8], p.1). They note how well understood problems in research ethics (i.e. vulnerability, voluntariness, undue influence, full disclosure, equitable subject selections, conflict of interest) become particularly problematic in the professional sports context, as opposed to the more typical health, medical and scientific contexts in and through which research is already governed.

In addition, the athletes-guinea pigs lack protection against the conflict of interest that can arise when the individual’s long-term health is not the goal of the innovations being introduced in the professional sport context, despite the presence of idealized statements in key medical pronouncements such as the International Olympic Committee’s 2009 Medical Code [9]. Quite to the contrary, short-term gains and the gaining of 'competitive edge’ are often in conflict with the long-term health of the athlete-subject [10-12].

In this essay we build on the analysis by King and Robeson, and apply the foil of Italian essayist Italo Calvino [13] to analyse the ethical issues of current modes of research participation. To this end we compare the two communities of human guinea pigs, whose juxtaposition helps bring into focus problematic issues in both practices. In his collection of essays “Six memos for the next millennium”, Calvino spelled out six 'values’ or qualities that he thought it was important to preserve in the transition to the next millennium: lightness, quickness, exactitude, multiplicity, visibility, and consistency [13]. For Calvino, these values pertained to the realm of literature and writing, but their value and significance need not be thus limited. In particular, three of these six values analysed by Calvino, namely visibility, multiplicity, and consistency seem particularly apposite to our analysis of contemporary practices of participation in research in professional sport and pharmaceutical research, and it is through these lenses that we will carry out our ethical analysis.

### Visibility

For Calvino, each lecture (or 'Memo’ as he put it) was to be devoted to the analysis of one indispensable literary value. Visibility dealt with the imaginative process, and was regarded by Calvino as a fundamental value, as it allowed readers to 'see things’, and to see the process through which that new seeing was enabled. In the context we analyse here, we understand 'visibility’ to mean two things: a) the level of information that individuals have access to in regard to the kind of drugs or pharmaceuticals they are being administered, or the regimes or surgeries they undergo; and b) the level of transparency, and thereby accountability, that characterizes the professional sport context.

Athletes lack information on the safety and effectiveness of the agents that they are taking, or of the performance enhancing technologies (e.g. injury prevention, training schedules, post-trauma surgeries) that they are subjected to. As a matter of fact, in the current system where innovations are translated directly into athletics amounting to 'unregulated clinical research’ ([8], p.1), the safety and effectiveness of the agents and methods cannot be adequately determined. The lack of information means that, in some cases, athletes take risks without experiencing any benefit. This happens because the WADA CODE does not require that a substance have a demonstrably performance-enhancing effect for it to be included on the Prohibited List, making the rational underpinnings of the decision to have a substance enter the List or not somewhat opaque. At present, it suffices that the substance has the 'potential’ to enhance athletic performance, in addition to meeting one of the other two criteria of the definition of doping: that it is harmful (or potentially so), or that it is against the spirit of sport [14]. We will discuss the WADA CODE and its criteria more in detail in Section The need for research on enhancements.

There is also a further sense in which the visibility of athlete guinea-pigs in the elite sports community is very limited: the data on safety and efficacy does not see the light of day in scientific journals, or does so in – one suspects – anodyne form and only after competitive advantage is not compromised. The opacity surrounding the science and medicine of elite sports is problematic since individuals and sport science teams do not welcome external scrutiny. Thus, complete transparency of novel interventions can undermine the very *raison d’etre* of sports science and technology support systems. A lack of transparency clouds the appreciation of lines of accountability, as we discuss in the case of Birgit Dressel below. Complete transparency of course undermines competitive advantage.

### Multiplicity

In literature, multiplicity refers to the infinite possibilities of intertwined reality and fiction open to humankind, in a kind of Russian doll multiplication of possibilities that we find in the works of Jorge Luis Borges, Carlo Emilio Gadda, and Calvino himself, among others. Multiplicity is a hidden feature of the world. While reality can of course be read as having a single layer of meaning, the discovery of hidden strata of significance demands an attentive interpreter of the world. In the context of professional sport and pharmaceutical research, multiplicity becomes also a central feature from the perspective of the subjects. In the context of pharmaceutical research, 'volunteers’ participate in more than one Phase 1 trial at a time, and are being administered a cocktail of drugs, as we discuss in Section Human guinea pigs in the pharmaceutical industry. In both cases, the multiple drugs being administered have n-possible combinations of interactions, the result of which is difficult, if not impossible, to predict.

German heptathlete Birgitt Dressel is an example of high dosage polypharmacy doping, or what we shall refer to after Calvino as “pharmaceutical multiplicity”. Birgit Dressel was born in Bremen, West Germany, in 1960. She came in ninth at the 1984 Olympic Games in Los Angeles, and fourth at the 1986 European Championships in Stuttgart, West Germany. She died on April 10, 1987 due to multiple organ failure caused by the combination of pharmaceuticals she had been ingesting over the past months aiming to enhance her athletic performance [15]. In response to her mother’s anxieties, Dressel is reported to have said “These are all harmless drugs. All athletes take them. It's really nothing special” [16]. An autopsy revealed traces of 101 different medications in her body including bovine tissue [17]. The investigation report on her death concludes that despite her powerful appearance Birgit Dressel was the opposite of a healthy person: as reported in the German newspaper 'Der Spiegel”, Dressel was “in truth a *chronically sick young woman* [emphasis added] pumped full with hundreds of drugs. Sport had made a cripple of her long ago, destroying her joints and ruining her internal organs prematurely” [18].

**Figure 1:**
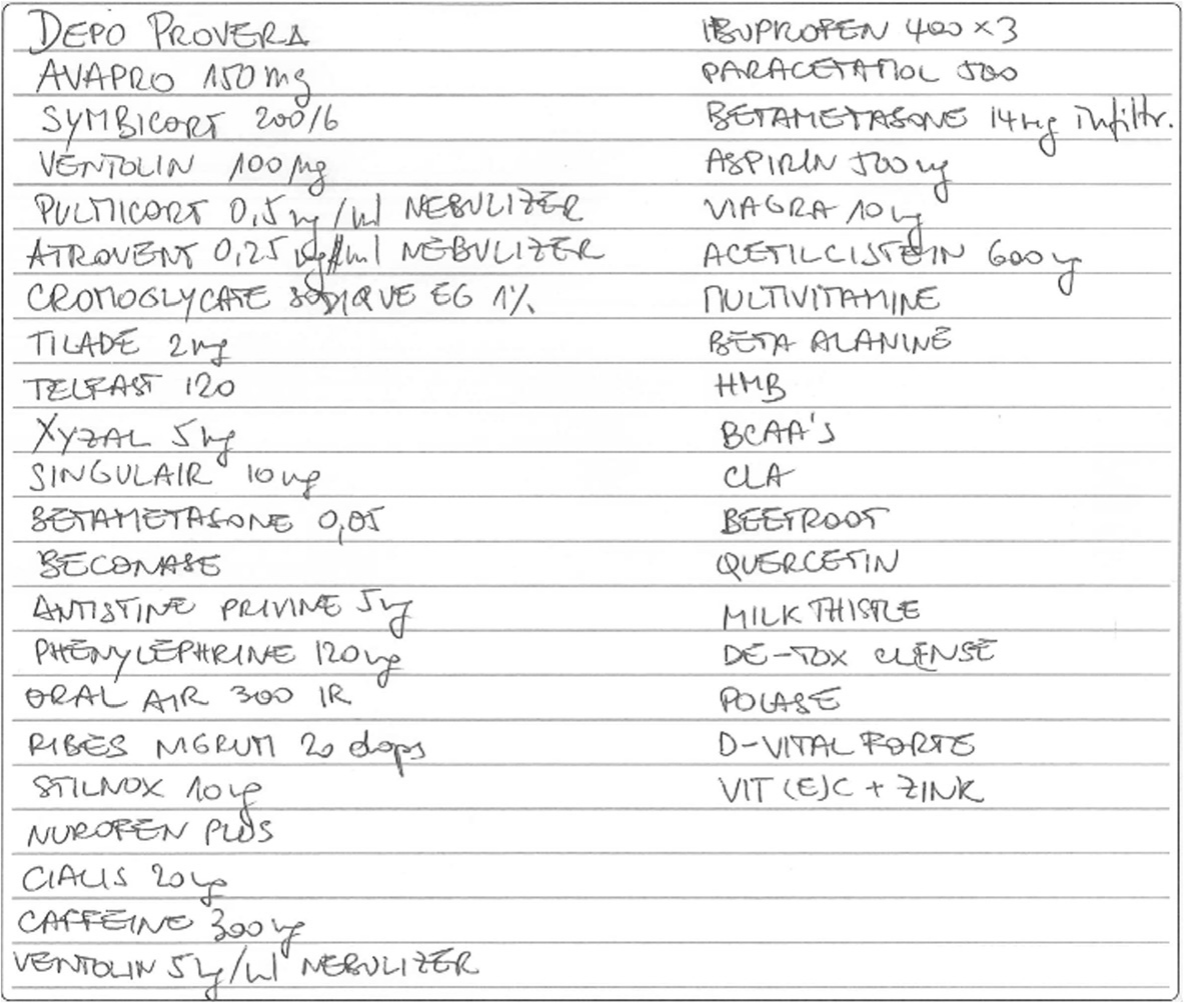
**Anonymised combination of drugs declared by a professional cyclist at a drug control.** Courtesy of UCI.

What is widely believed to be a further case of such pharmaceutical multiplicity is American track and field athlete Florence Griffith Joyner (Flo Jo as she was ubiquitously known) (1959–1998), who was known as the 'fastest woman of all time’, and whose career was dogged by rumours of drug abuses which linger even today as the causes of her premature death at 38 years old. Her autopsy, while not being conclusive as to the causes of her death, revealed the presence of a cocktail of drugs in her body [19]. Less dramatically, several of Lance Armstrong’s past professional cycling entourage have reported use of human growth hormones, testosterone and erythropoietin in combination [20]. The professional cycling world has been one of the athletic contexts where doping practices are most widespread, but the possibility of widespread multi-product doping is a reasonable assumption in some, though not all, sports, where athletes participating in doping practices expect to obtain 'enhancement benefits’ that would provide them with the competitive edge they are looking for to beat competitors, along with such extrinsic benefits (prize money, media profile, sponsorship, etc.) as come in train. Figure 1 illustrates the combination of drugs that a professional cyclist declared to be taking at a drug control visit (note that this quite extensive list accounts only for the drugs that he declared himself to be taking).

The combination of these substances, and those (if any) not declared, along with the extraordinary training schedules that athletes engage with, will bring about effects that cannot be fully understood nor controlled by those in the sports medical entourage. This fact is exacerbated as the data on the 'potential’ performance-enhancing effect of an agent are currently extrapolated from clinical trials conducted with therapeutic purposes, on a pool of subjects that has little in common with the population that will eventually be prescribed the drug (see Section The need for research on enhancements for a discussion of the problem of 'externality’).

### Consistency

'Consistency’ was to be the sixth and final lecture that Calvino prepared for his visiting professorship at Harvard, but that unfortunately he could not deliver due to his sudden and premature death in 1985. Here, we understand 'consistency’ in respect to the supposed integrity of sports, which athletes who engage in doping practices are said to be jeopardizing. WADA claims that: “In all of WADA’s work and the fulfillment of its Strategic Objectives, it will: preserve the integrity and value of sport and youth” [21]. Indeed, one of the three defeasible WADA criteria for a product or process to be considered for the Prohibited List is that it violates “the spirit of sport” [14]. How should we understand this claim?

In the contexts of professional sport, what threatens their integrity is the notion of something like a victor who has been tested in sufficiently similar ways with recourse to sufficiently similar modes of preparation and training [22]. Sports enshrine formal equality; once athletes are enjoined in competition they must submit to the same constitutive and regulative framework [23]. Fairness requirements in particular must be satisfied, though these are sometimes more discretionary (such as the size of the playing roster, the budget for the team, the drafting process, and so on [22]). Anti-doping policy is not, however, discretionary in this way. In wanting the best athlete or team to win, we understand that part of what “best” means is that the athletes or teams submit themselves to the formal and informal rules of their sport, and excellence within that framework. Considered analogically, if sports were to be understood as athletic experiments [24], then they should not be contaminated by extraneous variables (such as excessive luck, bribery and corruption, incompetent officiating, or judging, and so on). Doping, given the existence of global anti-doping policy, thus represents a perversion of athletic talent and a contamination of the integrity of the contest. Of course the analogy should not be pressed too hard. Luck, for example, is an element of all sport, though we can recognise how an excess of it can gift a lesser opponent a pyrrhic victory. Nor can one entirely equalize pre-competition support (whether scientific or economic). Nonetheless, sports legislators make considerable efforts to maintain the integrity of sports understood as the provision of an equitably balanced competition where there is fair opportunity for uncertainty of outcome to be a reasonable proposition. This is precisely what doping, though not exclusively, usurps or contaminates.

We cannot discuss here at length the objections to WADA retaining the 'spirit of sport’ criterion in the revised 2015 Code. It is frequently the target of an unsustainable objection that it is in principle conceptually vague – and therefore more open to abuse – than the performance enhancing and harm to health criteria. Not only does this objection fall foul of anti-essentialist philosophical objections, but it also ignores the literature that amply demonstrates the contestedness of the concept of health [25].

The recognition that athletes are guinea pigs in an unregulated or improper system of research prompts a reflection also on another problematic mode of participation in clinical research, namely healthy volunteers who make a living out of participation in Phase 1 studies, mainly but not exclusively in the USA. In the next section we highlight how, in a similar way to what happens in the context of professional sports, norms of research ethics are exacerbated in this new mode of participation in research.

## Human guinea pigs in the pharmaceutical industry

In the last few years social scientists have taken an interest in the new realities of 'professional guinea pig communities’ in the USA [26-30]. Elliott [27] and Abadie [28] document respectively from an ethical and an anthropological point of view the realities of the healthy individual making a living of participating in one Phase 1 trial after another, or even in more than one Phase 1 trial at a time. Phase 1 studies are aimed at testing safety of a new pharmaceutical drug or treatment in a restricted number of patients, after the treatment has proved efficacious in laboratory testing and animal models [31]. While in the early '90s more than 80% of industry-sponsored trials were conducted in academic health settings, by 2005 only 25% of all US clinical trials were conducted at universities, and this shift away from an academic setting to a pharmaceutical one is increasing [26]. As the US still conducts about 60% of all clinical trials worldwide, this represents a major change in the field of clinical research [32].

Who are these 'professional guinea pigs’? Typically the population comprises healthy young males in their 20’s and 30’s who belong to un (der) insured fractions of the US population, which can be easily recruited by the pharmaceutical industry into clinical trials in exchange for payments and limited medical attention during the study. Payments ranging from $100 to $300 per day are not uncommon [33]. As noted by Fisher (2009), some of the participants, being without [sufficient] medical insurance, see participation in clinical trials also as a way of having access to care [26]. This perception though is a misperception on their part, as even the limited medical attention that they may receive during the study is limited to the physiological parameters, which are a necessary condition for their participation. This perception, we argue, could be regarded as a 'variant’ of the widespread phenomenon of 'therapeutic misconception’ [34] that characterizes Phase 1 trials. Therapeutic misconception arises when subjects misinterpret the primary purpose of a clinical trial as therapeutic, and conflate the goals of research with the goals of clinical care [34]. By analogy, we could say that athletes who engage in doping practice without any evidential basis of the performance enhancing effects of the substances may also be victims of a similar phenomenon, which we refer to as the '*enhancement*’ where they think they are receiving a benefit (i.e. a performance advantage), while they are not. What both communities of human guinea pigs are receiving for sure are health risks. Adverse effects such as headaches, skin reactions, nausea, and diarrhoea are very common in Phase 1 trials. More serious risks like allergic reactions, liver/kidney failure, and severe arrhythmias are rarer, but by no means unique. In limited cases death is the outcome of research participation [35]. We noted in Section Multiplicity the non-trivial health risks that athletes are willing to take in exchange for the hope of a performance enhancement.

While it may seem difficult to accept that one and the same participant can be enrolled in multiple trials at the same time, or successively in one trial after the other, this practice is made comprehensible by the economically driven reluctance of pharmaceutical companies to share information about participants with their rival companies. Thus, insofar as the volunteers themselves do not disclose their other commitments and are able to juggle their schedules, they can be enrolled in more than one study at a time run by different companies. It is quite obvious therefore that the current system of clinical research as run by pharmaceutical companies rewards a high-risk attitude among serial participants, as it incentivizes them not to respect the normal wash-out period of thirty days in order to be enrolled in as many trials as possible.

In research ethics, 'vulnerability’ is typically understood as the participant degree of (in) competence to reach an autonomous decision regarding participation in clinical research, after having assessed - free from coercion or undue influence - the available alternatives [36]. Being research subjects in an unregulated research system, as the professional sport context, makes athletes 'vulnerable’ subjects, and therefore in need of extra layers of protection that, as we argue below, would take the form of a proper framework for research on performance enhancing methods and substances. It seems therefore that serial participants in early pharmaceutical research could also qualify as vulnerable from a social and economic point of view [36], and as such would deserve special protection that they do not currently enjoy. In addition, participants are incentivized not to disclose any adverse events they might be experiencing: the burden of proof to demonstrate the causal link between the clinical trial participation and the adverse event is on the participant, and dropping out of a trial compromises the ability of the individual to remain a serial participant, and jeopardizes the significant financial 'completion-bonus’.

For these reasons, the practice of serial participation in Phase 1 trials is seriously problematic from an ethical point of view, and highlights issues that are present also in the context of professional sport. Indeed, both the current system of clinical research as run by pharmaceutical companies and of professional sports reward high-risk behaviour and lack of transparency among serial participants. Both communities of serial participants can be considered vulnerable on different - though related - grounds. Both communities also lack visibility, in the sense of lack of systemic accountability, due to the absence of a central regulatory system (in the context of pharmaceutical research, individuals can participate in multiple studies run by different companies that do not share data on their pool of participants), and in the sense of lack of information that the subjects have on the substances they are taking, and the procedures to which they are subjected. Finally, both communities face a problem of consistency, as the effects of financial incentives not to disclose adverse events, not to respect the required one month wash-out period between trials, and not to comply with the study requirements, further cloud a precise and proper appreciation for the potential enhancing effects of the agent and its side effects.

We believe that the combination of these factors renders near-impossible sound inferences on the effects of the pharmacological agent, and that a regulated research system aimed specifically at studying the performance enhancing effects of agents is urgently needed. To this problem we now turn.

## The need for research on enhancements

The only existing, albeit small, review of the few existing studies with substances that might enhance athletic performance has been conducted by Maschke (2009) [37], who points out the tension raised by the necessity to research on the performance enhancing effects of such agents, together with the necessity to develop accurate detection tests on the right pool of subjects, and the legitimization that such necessary research would seem to impress on the use of doping agents. Green (2009) further illustrates the point of “externality” (noted in Section Human guinea pigs in the pharmaceutical industry above), or of the unrepresentativeness of the subjects of the study [38]. He writes of his participation in a study aimed at validating testing to detect recombinant erythropoietin (rHuEPO) (a doping product used to enhance speed endurance) before the 2002 Winter Olympics. Green notes that one of the criteria for inclusion of volunteers in the study was that the subjects could not be subjected to drug testing, nor actively competing at a level that would render them a potential subject in a relevant anti-testing pool (this in accordance with WADA Code). Under these circumstances, Olympic athletes who commit an anti-doping rule violation (i.e., return a sample that is positive for substances) could challenge the validity of the testing, on the basis that the test was not developed with a representative cohort, and that somehow Olympic athletes metabolized rHuEPO differently from untrained individuals [39].

The rHuEPO case should not be understood as an isolated example, as the problem of the reliability of the extrapolation of data obtained on a pool of healthy volunteers (and in addition, who participate in several trials at a time, or in one trial after another) to a pool of elite athletes is much broader, and encompasses all the doping substances that are on the WADA banned list, plus the many other agents which may have potential performance-enhancing effects and that have not been included yet in the WADA list. As Eynon points out, it is problematic that: “Much of what we already know on sports genetics and will learn in the future has to be inferred from studies in non-athletic populations” [39]. In so far as there is disagreement as to the extent that valid and reliable methods of detection exist, athletes who test positive may challenge the scientific validity and reliability of results obtained on a different pool of subjects, with different doses of substance, or not obtained at all. This contestation speaks directly to the perceived legitimacy of anti-doping policy.

Even if we recognize the tension pointed out by Maschke [37], it does not follow that when one argues for research on enhancements one thereby legitimizes or endorses the enhancements one is researching on. Rather, we hold that an analysis of the ethical permissibility of research on enhancements should proceed with a two-tiered strategy, by focusing first on how to justify such research at a general level, and then on analysing the ethical permissibility of a particular research on a case-by-case basis [40]. As to the latter point, what are these ethical requirements that research on enhancements should fulfill in order to be permissible? It is commonly accepted that for clinical research to be ethical, it must fulfill seven individually necessary and jointly sufficient requirements: It must (1) have health-related social value, (2) be scientifically valid, (3) use fair subject selection, (4) involve a favorable risk-benefit ratio, (5) be independently reviewed, (6) satisfy informed consent requirements, and (7) respect enrolled participants [41].

How might these requirements translate to research on enhancements? The translation of criteria (2) to (7) from a clinical research context to an enhancement context seems to us to be quite straightforward. On the contrary, the translation of criterion (1) is not straightforward. It is not immediately clear that research on enhancement ought to be justified by having health-related social value, even though there might be some cases of 'dual use’ biomedical interventions, or interventions that can be used both as treatments and as enhancements. In such cases any health-related social value can be seen as an added value rather than a prerequisite. The health of the athlete should of course still remain a primary concern (see below for a discussion of the risks/benefits evaluation), but research on performance enhancing substances should not have as one of its goal the promotion of health. Its first epistemic goal pertains to the validity and reliability or otherwise of performance enhancement claims. Of course this epistemic goal is framed by an ethical one. Thus the evaluation of risks and benefits (criterion 4) needs also to be modified when shifting from the clinical to enhancement contexts. Precisely what counts as benefit and risk in enhancement research need not be identical with what counts as benefit and risk in clinical research.

In addition to the criteria spelled out by Emanuel et al. (2000) for the justification of clinical research [41], it is necessary to add a criterion of 'accountability’ to the justification of enhancement research, both in clinical and enhancement contexts. This would increase the transparency, or visibility using Calvino’s foil, of two practices of research described in this essay. As illustrated above by Dressel’s case, the lack of accountability is a widespread problem in professional sport, and the absence of a central regulation allows and incentivizes high risk behaviour of serial participation among health volunteers in Phase 1 trials.

Let us assume for the moment that research on enhancements can be justified from an ethical point of view at a general level, on the basis of the criteria spelled out above. To reiterate, we do not argue that this first level of justification would imply that research on enhancements *tout court* would also be justified: individual cases would still need to be reviewed and justified –or not – on a case-by-case basis. Indeed, there has been considerable disquiet in the anti doping policy world about the adequacy of the evidential basis (or lack thereof) that WADA use to base their decisions for inclusion of substances and methods (i.e., what constitutes doping) on their Prohibited List. During the recent second revision of the WADA CODE, there was considerable support to alter the criteria for choosing items on the prohibited methods and substance list (what most people understand to be designated by the term 'doping’). Revisionists argued that the 2015 WADA CODE should elevate 'performance enhancement’ from being merely one of three defeasible criteria to the sole necessary condition of doping, to be supported by either of both of the remaining (now) secondary conditions: '(potential) harm to health’ and '(potential) contrariness to the spirit of sport’ [25]. This would increase the visibility of the substances in the Prohibited List, which at present, are not (or at least often not) supported by high quality evidential bases [37]. Indeed, many of the substances included in the Prohibited List are merely *presumed* to be ergogenic, and have not been subjected to rigorous, randomized controlled studies, or if they have, that was done in a different pool of subjects than those who are effectively going to take the substances.

The inclusion of the performance enhancement criterion as a necessary condition in the revised 2015 WADA CODE was widely supported during the revision process, and yet it was rejected in the final version of the revised CODE. Nevertheless, if the performance enhancing criterion indeed were to have become a necessary condition, there would need to be reasonable grounds for a substance’s or method’s inclusion on the Prohibited List – reasonable grounds that, at the moment, do not appear to be evident. Therefore, we have argued in this paper that a proper governance framework needs to be established, both to assess the performance enhancing effects of the substances, and the risks of harm to the health of the athlete. We note here the problematic nature of the 'potential risk to the health of the athlete’ criterion, which, contrary to the potentiality of the performance enhancing effect, has not been under discussion in the Code revision process. Further discussion of this problem is beyond the scope of the present essay (see instead [25]).

We do not, by any means, argue in favour of WADA suspending their enforcement power until a certain point in the future is reached when the performance enhancing effects of each of the substances or methods on the Prohibited List is demonstrated. We argue instead that the inclusion of the substances in the List should proceed following the precautionary principle [42], though it should not be used indiscriminately [43], but only where there are reasonable grounds to infer a performance enhancing effect, or risk to the harm of the athlete^a^. Sometimes this will involve reasonable extrapolation. For instance, there is good evidence to suggest that the use of beta-blockers enhances performance in pistol shooting [44] and that this might be contrary to the spirit of sport. Thus beta-blockers are banned. Yet the ban applies not only to target sports - where the inference is reasonable - but also to other sports including Formula 1 style motor racing, skiing and snowboarding, where its performance enhancing effects seem less than obvious. To be clear, we would not count as reasonable grounds anecdotal evidence on performance enhancing effects or harmful effects a substance, such as creatine with (say) a particular population (children).

We know as a matter of fact that elite athletes will take risks. A century ago that would have included the use of strychnine [45] in what we referred to as the enhancement and a more scientific approach to performance enhancement may well have fueled the use of growth hormones [46,47]. Yet, the precise enhancing effects here are not clear. Is the inference so obvious that it invokes the precautionary principle? Probably: but transparency would require a reasonable and public statement of the principle. Sadly, WADA have not hitherto been minded to operate in this way and we suggest that compliance and concordance with the WADA Code might follow upon greater visibility as to their decision-making.

## Conclusions

As noted by King and Robeson (2007) [8], the translation of norms of medical ethics to sport medicine without their re-visioning can be problematic. The clash of interests in sports medicine between services to the athlete-patient and the contracting party is well known [3], but discussions are more frequently framed in terms of return to play issues or enhanced recovery. In this paper we have focused on the relatively unexplored nexus of medical ethics, research ethics and sport medicine, using as a case study two communities of human guinea pigs: the athletes and the serial participants in Phase 1 studies.

As our analysis has shown, both doping and phase 1 trial scenarios are similar in the *multiplicity* of the drugs that the individual is simultaneously taking; the lack of *visibility* (in the sense of transparency and accountability) of the context; and the high level of risks the individuals engaged in these practices are taking onto themselves. The benefits expected for the participants differ in nature and magnitude, but the risks both communities are engaging in are clear. Both scenarios also pose a problem of *consistency* both in regard to the application of the trial guidelines, and of the value of the data that are extrapolated from such research. Thus, both categories - although for different reasons- can be considered vulnerable and worthy of special protection through the establishment of a proper governance framework for regulation of research.

The practice of professional guinea pigs in Phase 1 trials represents an issue of exploitation of economically vulnerable subjects in a developed society (USA) that has allowed the pursuit of medical and scientific knowledge to move away from an academic setting to a pharmaceutical industry concerned only with profit and that, under a rhetoric of participation and progress, exposes the individuals to a multiplicity of agents whose interactions are unknown and impossible to infer, in exchange for a limited paycheck whose payment schedule incentivises high-risk behaviours^b^.

Returning to Calvino’s foil and the context of professional sports, we propose the legitimization of a research enterprise to measure the enhancement effects of agents within a systematic governance framework. This would promote greater visibility and consistency in the context of professional sport currently lacking both, and where athletes may be subjected to an unregulated research system, which poses high risks to their health. This might also substantially increase the visibility of the substances in the Prohibited List, which at present are not (or at least mostly not) supported by high quality evidential bases. If the performance enhancing criterion were to become a necessary condition in a subsequent version of the WADA Code, then there must be greater justification and transparency concerning the grounds for the inclusion of substances in the Prohibited List. There also need to be reasonable evidence that the substance is harmful to health as opposed to an indiscriminate use of the precautionary principle.

As for multiplicity, it is a feature of our millennium that we regard as impossible and undesirable to be rid of. Nevertheless we can, and should, devise better ways to navigate it.

## Endnotes

^a^This blanket application of the precautionary principle is evidenced by WADA’s category of Non Approved substances. Prior to specifying particular products or processes they write: “Any pharmacological substance which is not addressed by any of the subsequent sections of the List and with no current approval by any governmental regulatory health authority for human therapeutic use (e.g. drugs under pre-clinical or clinical development or discontinued, designer drugs, substances approved only for veterinary use) is prohibited at all times.” [http://list.wada-ama.org/prohibited-in-competition/prohibited-substances/] Accessed 31.10.2013.

^b^Rather, we believe that the pursuit of medical and scientific knowledge should not solely be left in the hands of the pharmaceutical industry, but we cannot pursue this line of inquiry in depth here. We refer to Fisher for an insightful analysis of the economic, social and political milieux of the shift from research conducted within the academic realm to the private sector [26].

## Competing interests

The authors are not aware of any competing interests.
